# Technical standards for recording and interpretation of neonatal electroencephalogram in clinical practice

**DOI:** 10.4103/0972-2327.48869

**Published:** 2009

**Authors:** Perumpillichira J. Cherian, Renate M. Swarte, Gerhard H. Visser

**Affiliations:** Departments of Clinical Neurophysiology, Erasmus MC, University Medical Center, Rotterdam, Netherlands; 1 Departments of Neonatology, Erasmus MC, University Medical Center, Rotterdam, Netherlands

**Keywords:** Amplitude integrated EEG, electroencephalogram monitoring, hypoxic ischemic encephalopathy, neonatal electroencephalogram, neonatal seizures

## Abstract

Neonatal electroencephalogram (EEG), though often perceived as being difficult to record and interpret, is relatively easy to study due to the immature nature of the brain, which expresses only a few well-defined set of patterns. The EEG interpreter needs to be aware of the maturational changes as well as the effect of pathological processes and medication on brain activity. It gives valuable information for the treatment and prognostication in encephalopathic neonates. In this group, serial EEGs or EEG monitoring often gives additional information regarding deterioration/improvement of the brain function or occurrence of seizures.

## Introduction

Electroencephalogram (EEG) offers a window to the brain function and has a unique place in the care of sick newborn babies. Its value is increased when neurological examination is confounded by the use of sedatives and neuromuscular blocking agents (even though EEG itself may be influenced by sedatives). It is done in a neonate commonly for the following indications: a) assess the severity of brain dysfunction, b) detect (subclinical) seizures, c) assess cerebral maturation, and d) determine prognosis.

To derive maximum benefit from an EEG, the neurologist needs to be familiar with maturational changes as well as the effect of various pathological conditions on the EEG. Often, the recording has to be made in the NICU, with interference from monitoring equipment, indwelling lines, incubator, and frequent nursing care. In this article, we highlight those technical details that are relevant for neonatal EEG registration and describe normal and abnormal EEG phenomena in the preterm and the term neonate.

## Recording the EEG: Preparation, precautions, and technical aspects

### Patient information

#### Age of the neonate:

Knowledge of the exact age of the baby undergoing EEG is important for a proper interpretation. Maturational changes occur fairly rapidly in the 25–48 week period [Table 1] and any discrepancy in the age-related EEG findings of more than two weeks is abnormal. The gestational age (GA) is defined as the time elapsed between the first day of the last menstrual period of the mother and the birth of the baby.[1] A baby born prematurely at 32 weeks and having a chronological age of four weeks at the time of EEG recording is considered to have a postmenstrual age (PMA) of 36 weeks. This corresponds to a conceptional age (CA) of 34 weeks, assuming that conception occurred two weeks after the first day of the last menstrual period. However, in practice, the term CA is used interchangeably with PMA by many authors.

**Table 1:** Salient normal electroencephalogram features at different conceptional ages

**Age (in weeks)**	**27–28**	**29–30**	**31–33**	**34–35**	**36–37**	**38–40**	**41–44**
**EEG feature**							
Major characteristics (background activity)							
Tracé discontinu	+++(>80%)	++	+ (50%)	+/-	-	-	-
(discontinuity, seconds)	10–30	8–15	6–10	2–6		
SATs high amp	+++	++	+	+	+/-	-	NA
complexity	+/-	+	+	++	+++	+++	
Sleep–wake	-	+/-	+	++	+++	+++	+++
cycles				AS 60%		AS 50%		
Tracé alternant	-	-	-	+	+	++	+
Tracé continu	-	-	-	+	+	+	++
Occurrence				(W, AS)	(W, AS)	(W,AS,SQS)	(W,S,SQS)
Synchrony	++	+/-	+	+	++	++	+++
(of EEG ‘bursts’)	>80%	<50%	>50%	>70%	>80%	~100%	100%
Minor characteristics (paroxysmal wave forms)							
Delta brushes	+/-	++	+++	++	++	+	+/-
Location		C	TO	TO	TO	O	
Occurrence			W, AS,QS	W, AS,QS	QS	QS	
Theta activity	+	++	++	+	+/-	-	-
Location, predom	O	T,O	T,O	T,O			
Delta activity	-	+(AS)	+(AS,QS)	+(QS)	+(QS)	+(QS)	+(QS)
Location, predom		O	O,F	O,F	F	F	F
Frontal sharp waves	-	-	+	++	+	+/-	-
Sporadic sharp waves	+/-	+	+	+	+	+	+/-

Tracé discontinu: Quiet periods show a voltage <25 μV (often < 10 μV); SATs: spontaneous activity transients, intermittent slow transients seen in prematures; high amp: higher amplitude (>100 μV), complexity: consisting of waves of multiple frequencies; Quiet sleep (QS): consists of TA and SQS; Tracé alternant(TA), part of QS: Quiet periods of >25 μV, alternating with bursts of 100–200 μV; Active sleep(AS or REM): differentiated from wakefulness (W) by absent muscle tone in chin EMG channel, rapid irregular eye movements in EOG channel, and irregular respiration. AS decreases with maturation; Tracé continu: Irregular delta and theta of 50–100 μV during wakefulness (W) and AS, High amplitude (>100 μV) delta during slow quiet sleep (SQS); Delta brushes: rhythmic beta activity superimposed on delta waves. Amplitude decreases with maturation; Theta activity: temporal and occipital saw tooth waves, 4–7 Hz, 0.5–1.5 seconds in duration; Predom: predominant; C: central; O: occipital; T: temporal; F: frontal; Frontal sharp waves: encoches frontales; Sharp waves: occur sporadically in different locations, predominantly central, 1–4/minute

#### Clinical information:

Time of birth, history of birth asphyxia, Apgar scores, occurrence of convulsions, etc. need to be noted by the technician before starting the recording.

#### Medication use:

Medications like morphine, barbiturates, and benzodiazepines may influence the EEG findings,[2] especially by lowering the voltage of the background activity. Their dosages, time of administration, and serum levels, if known, should be noted.

#### State of the patient:

Noting the condition of the neonate, whether awake or asleep, on a ventilator, lying in an incubator, etc. is relevant. This helps not only in relating EEG phenomena to the state of the patient, but also in recognizing artifacts like those arising from high-frequency ventilation. Changes happening in the environment like loud noises, flashes of bright light, nursing care, etc. should also be noted, as these may produce transient attenuation of the background activity or produce movement artifacts.

### Recording the EEG

#### The timing of registration after birth:

If the EEG is done to assess the degree of brain maturation, it can be done at least 24 hours after birth to ensure that transient EEG abnormalities caused by birth itself are not recorded.[3] If however, the goal is to assess the degree of encephalopathy and detect subclinical seizures, in situations like perinatal asphyxia, it is advisable to start the EEG registration (as a part of serial EEGs or EEG monitoring) as early as possible (at least within 24 hours), after respiratory and hemodynamic stabilization.

#### The duration of recording:

Recording with the child asleep as well as awake is needed for the proper interpretation of neonatal EEG. A record made after feeding the baby is likely to succeed in this. The preparation for recording, like pasting of electrodes, can be done toward the end of the wake period so as to ensure that the neonate is not disturbed during sleep.[3] The registration time should be 45 minutes or longer. Abnormal or absent sleep-wake cycling[4–6] is sometimes the earliest indicator of brain dysfunction, and in such situations, it is required to record the EEG for a longer period (at least two to three hours).

Adequate skin preparation is required to achieve a scalp impedance of <5 kΩ. Mildly abrasive pastes (like NuPrep™ gel) applied using a cotton bud, followed by alcohol swabs generally give a good result. Enough time (at least 90 minutes) has to be scheduled for the EEG recording to avoid stressing the technician and the parents.[3] This is preferable to registering an artifact-filled EEG, and later trying to minimize their influence, for instance by changing the filter settings.

### Electrodes and montages

At our center, we use the full 10–20 system of electrodes for all neonatal EEGs done during working hours and the restricted system of electrode placement during emergency EEGs done outside the working hours. The restricted 10–20 system of electrode placement uses nine active scalp electrodes – Fp_1-2_, Cz, C_3-4_, T_3-4_, and O_1-2_ electrodes.[3] Important spatial information is lost by using lesser number of electrodes. However, most clinical indications for an emergent EEG at this age do not call for a high degree of spatial resolution. Assessment of background activity is not affected by the reduced number of electrodes. The reduced montage has been shown to have a high sensitivity (96.8%) and 100% specificity when compared to a full 10–20 montage in detection of neonatal seizures.[7] Silver–silver chloride EEG electrodes with conductive adhesive electrode paste (like 10–20™ paste) are used. In addition, at our center, we prefer using 3% collodion with small pieces of cotton or gauze for optimal fixation of the electrodes. This gives a better quality registration and also ensures that there is no deterioration of electrode contact if the recording has to be extended to continuous EEG monitoring. In a period of more than 25 years of using this method, we have not encountered any allergic skin reactions or hazards due to flammability. Compressed cold air is used for drying. Fire hazard due to the flammable collodion has to be borne in mind, and a hair dryer should never be used. Removal of the electrodes is done using an acetone-free solvent (we use collodion-remover from Mavidon™ Medical Products). Acetone works equally well, but its fumes are quite irritating. We use the same technique in a baby lying in an incubator. However, the incubator is kept open for sometime after fixing the electrodes.

**Figure 1:**
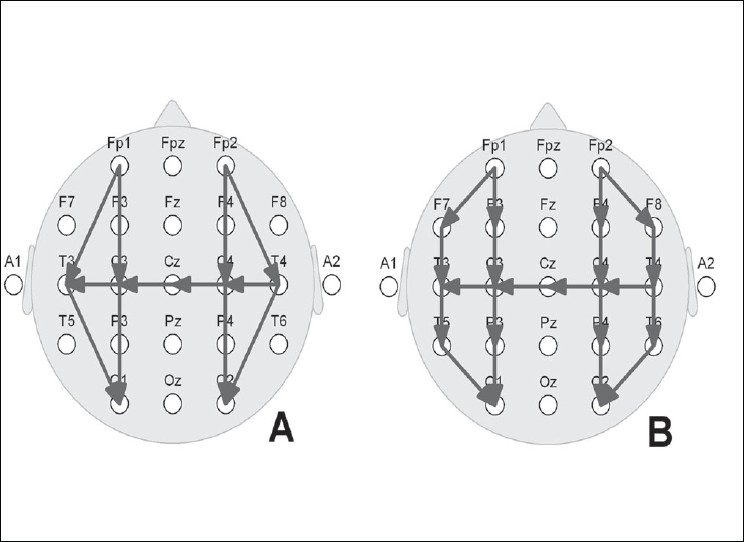
Neonatal EEG montages. A: Restricted 10–20 system using nine electrodes; B: Full 10–20 system of electrode placement using 17 electrodes. Note the bipolar derivations: anteroposterior right and left, as well as a coronal run including the central midline

Many EEG transients have a preponderance at the vertex, and hence, inclusion of the Cz electrode as well as a coronal bipolar electrode derivation in addition to the bipolar anteroposterior derivation is meaningful [Figure 1]. With digital EEG equipment, montage selection during registration of EEG is no longer very important.

#### Electrode caps:

They help to save time, especially when a full 10–20 system of electrodes, or a more extensive recording (e.g., high-density EEG) has to be made. They also help to keep the electrode positions fairly accurate, especially in centers where there are no experienced technicians. The quality of the record depends on a snug fit, and thus, a wide range of cap sizes fitting different head sizes would have to be available. The electrode positions may show some change over time and with repeated use, much more than with conventional electrode application.[8]

#### Filters:

Very slow waves (0.2–0.5 Hz) occur in the neonate and hence the low-frequency filter is set at 0.005–0.01 Hz. This very low filter setting may make the EEG more vulnerable to slow artifacts like sweat potentials. The high-frequency filter is set at 70 Hz.

#### Polygraphy:

Polygraphic registrations are the rule while recording neonatal EEG[3]. Simultaneous recording of video is also becoming a standard practice. Video helps in assessing clinical seizures and in recognizing artifacts. Physiological variables like eye movements, ECG, breathing pattern, and muscle activity maximize information about different stages of arousal or sleep, as well as paroxysmal phenomena like seizures. Eye movements [EOG channel] are recorded using two surface electrodes, placed diagonally, one centimeter above and below the outer canthus of the left and right eyes. Muscle tone is registered using a surface electrode placed over the submental region (chin EMG). ECG is recorded by two electrodes kept over the right and left side of the anterior chest wall. Another option is to place these electrodes over the right and left arms, in which case they can, in addition to the ECG, also record the limb movements. Extra channels for registering movements (transducers or surface EEG electrodes) may be added, for instance in the presence of clonic movements, tremor, hiccups, etc. Respiration is monitored by a transducer kept over the abdomen or the chest. Cessation of breathing for 6-7 seconds may be normally seen in neonates[9,10] and are considered to be pathological apneas meriting treatment only if they last longer than 10 seconds or occur frequently, or are associated with hemodynamic disturbances (like bradycardia or desaturations). Healthy premature infants may show short apneas as well as episodes of periodic breathing.[11] Other polygraphic variables like nasal airflow (measured using a thermocouple) and oxygen saturation (pulse oximetry), if available, may add important information.

### Reactivity

Transient attenuation of the EEG to external stimuli should be noted by the technician. Photic stimulation usually does not elicit photic driving in the term neonate and hence need not be done as a routine. However, in the premature infant, a striking photic driving may sometimes be observed, especially with lower flash frequencies.

## Description of normal EEG phenomena

### Maturation of EEG in the premature infant

**Figure 2:**
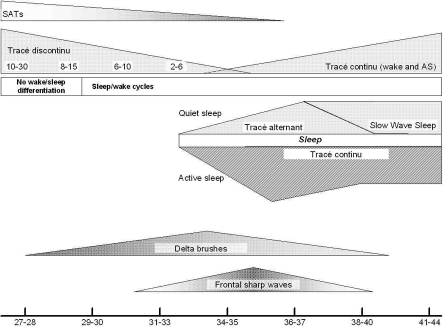
Scheme illustrating important maturational changes in the neonatal EEG, Tracé discontinu: duration of discontinuity is given in seconds. At the bottom, conceptional age in weeks

Literature describes EEGs recorded in prematures born as early as 23 weeks. The baby is considered to be premature till 37 weeks CA. EEG maturation is summarized in Table 1 and Figure 2.

### Major characteristics

Background activity

#### Discontinuity:

The hallmark of the premature EEG is discontinuity of the background activity (tracé discontinu, TD). This is characterized by periods of relative quiescence (voltage <25 μV, sometimes <10 μV), interrupted by bursts of EEG activity. The interburst interval (the length of discontinuity) shows a significant relationship with CA, with the periods progressively shortening with increasing maturation of the brain and development of cortical folding.[12] In addition, with maturation, the ongoing oscillatory EEG activity during the quiescent periods gradually increases in amplitude (becomes >25 μV by 36–37 weeks) and frequency, ultimately resulting in a continuous EEG trace at term.[13,14] The voltages are dependent on the montage used, and are given here as a rough guide for the EEG interpreter to help in classifying the records.

#### Spontaneous activity transients (SATs):

The term SATs has been used to describe the slow EEG activity during the bursts,[15,16] taking into account the similarity seen between this and the EEG activity in newborn animals like mice and rat.[17] Parallel with brain maturation, the amplitude of SATs decreases and their duration increases [Table 1 and Figure 2]. Their morphology becomes increasingly complex, with occurrence of waveforms of different frequencies. They usually disappear by 40–42 weeks. Delta brushes (low-amplitude faster frequencies superimposed on high-amplitude slow waves) are probably components of the SATs.[13,17] SATs in the developing somatosensory cortex are driven by early motor activity and may be important for normal development.[18]

#### Sleep–wake cycles:

Changes in state can be detected as early as 27 weeks, but it is only by about 31 weeks that differentiation of sleep–wake cycles (as detected by conventional short-duration EEG registrations) becomes well established. Awake EEG and active sleep (AS or rapid eye movement sleep, REM) are similar in morphology, in showing a continuous trace consisting of mixed frequencies, but can be differentiated using polygraphy. REM seen on EOG is the hallmark of AS, while wakefulness shows both rapid and slow eye movements. Both show irregular respiration but the (chin) EMG shows very little or no activity (hypotonia) with occasional brief bursts of activity in AS while it shows continuous activity when the baby is awake. Movement artifacts are more frequently seen in the wake EEG. AS may also show anterior slow dysrhythmia at 36–37 weeks. These are short bursts of delta activity occurring over the frontal regions.[14]

### Minor characteristics

#### Paroxysmal waveforms:

These include delta brushes, premature theta in the temporal and occipital regions, biphasic frontal sharp waves (*encoches frontales*), and sporadic sharp waves. Their location and frequency of occurrence varies with CA [Table 1]. Sporadic sharp waves of simple morphology are also seen, at 1–4/minute, varying in their location, more often in the central regions. Frequent sharp waves as well as sharp waves with complex morphology (‘polyspikes’), occurring rhythmically or consistently over a focus are considered abnormal. Their association with epileptic seizures is weak, and they are not termed ‘epileptiform’ at this age. Positive sharp waves in the central[19] and temporal regions[20] are considered abnormal and have been described to have varying association with pathological conditions like intraventricular hemorrhage and periventricular leukomalacia.

### EEG in the healthy term neonate

#### Awake EEG:

**Figure 3:**
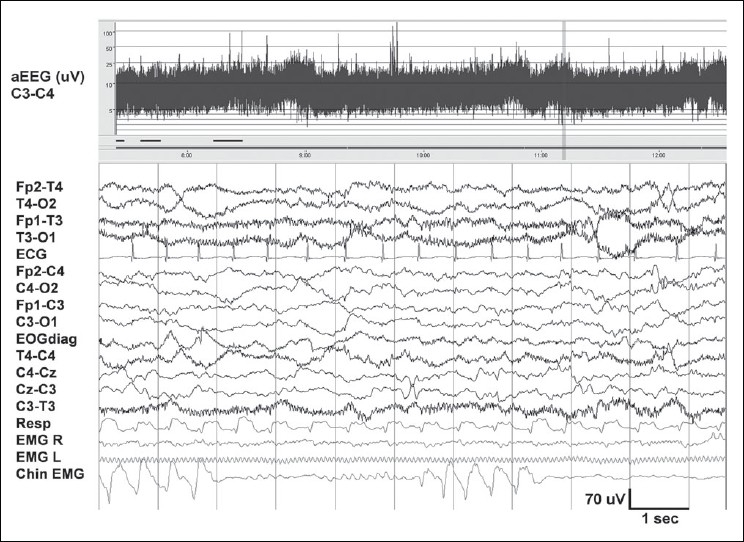
Awake EEG at term. Shows theta and delta activity with normal voltage (20–70 μV). The faster frequencies (>50 Hz) seen in the temporal channels are due to muscle artifacts. Chin EMG shows rhythmic artifacts produced by sucking. At the top, aEEG trend for a period of five hours. The band width between 5 and 20 μV is normal, and the fluctuations suggest sleep–wake cycling. The 12-second raw EEG sample in the lower part corresponds to the vertical bar on the right of the aEEG

Consists of continuous mixed frequency activity (*activité moyenne*) comprised of theta and delta activity of 70–100 μV [Figure 3], with the slower frequencies having a preponderance over the posterior head regions.[14] Short runs of theta activity of 1–3 seconds, and about 50 μV amplitude, are seen over the central region. The faster rhythms (4–8 Hz) also tend to dominate over the frontocentral regions.[21]

#### Sleep EEG:

Sleep periods often start with AS and it constitutes about 50% of the total sleep time. The EEG in AS shows continuous activity in all frequency bands with an amplitude of 50–70 μV. It is similar in morphology to the wake EEG and differentiation is made only with information from polygraphy.

**Figure 4:**
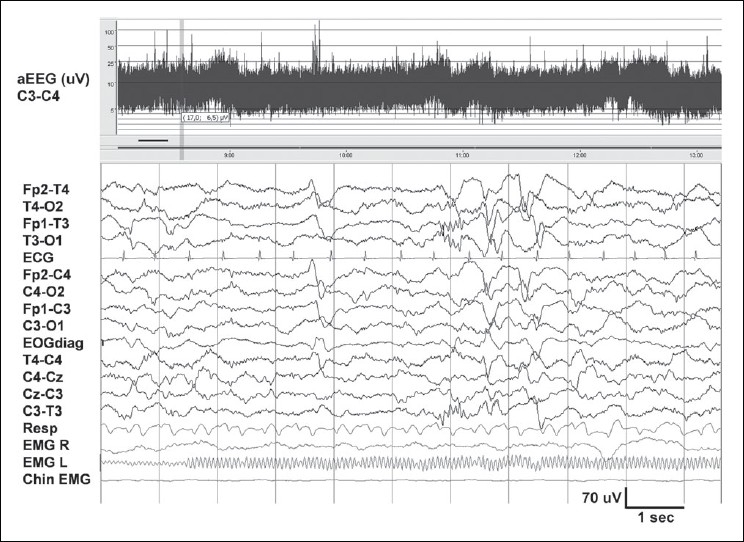
Normal quiet sleep in the neonate in figure 3. Tracé alternant with lower voltage during 2–5 seconds, and higher voltage in the next 3 seconds. Frontal sharp waves (encoches frontales) are seen at 4 seconds and brush-like activity between 6 and 7 seconds, T3-O1. Compared to figure 3, amplitude is higher and there is less muscle activity

Two types of quiet sleep (QS) are identified: tracé alternant (TA, an alternating pattern of ‘bursts’ and relatively quiet periods [Figure 4]) and slow QS.[14] TA is reminiscent of the discontinuous EEG of the premature. However, it shows activity higher than 25 μV (usually 40–50 μV) during the quiet periods. The ‘burst’ periods show theta and delta activity of up to 200 μV. Both periods last for 3–10 seconds. Slow QS consists of continuous high-amplitude delta activity over all regions. Frontal sharp transients (*encoches frontales*) are seen more during QS than AS.[22] Respiratory movements are regular in QS. However, using the above information, the sleep stage cannot readily be classified in about 20% of the situations, and may be termed as ‘transitional sleep’ or ‘indeterminate sleep’.[3]

## Abnormal neonatal EEG activity and its interpretation

Abnormalities in the neonatal EEG background activity and occurrence of excessive paroxysmal activity (both transients as well as epileptiform abnormalities) is seen in many encephalopathies like hypoxic ischemic encephalopathy (HIE).

### Abnormalities in background activity

#### Sleep–wake cycling:

One of the earliest abnormalities may be an abnormal or absent sleep–wake cycling.[5] This can be readily detected by trends like single channel amplitude integrated EEG (aEEG).[6] Even in relatively healthy neonates, sleep–wake cycles may be disturbed due to effect of medications or disturbances in the external environment as is often found in the NICU. Conversely, the presence of sleep–wake cycles in the neonatal EEG is a good prognostic sign.

#### Discontinuity and other abnormalities of voltage:

**Figure 5:**
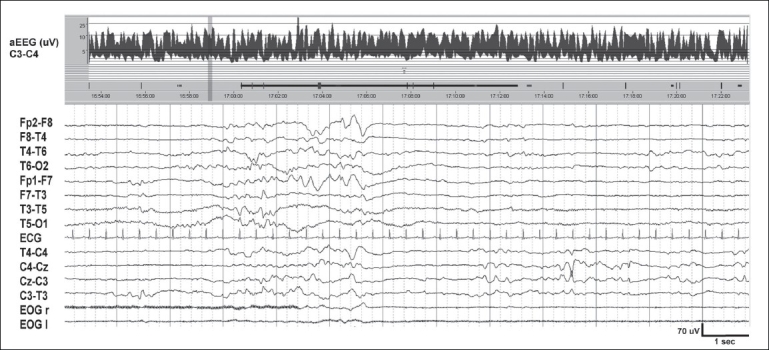
Trace discontinu in a neonate with moderately severe HIE. Background activity is of low voltage (<20 μV). The discontinuous periods, with a mean voltage of 10 μV, are interrupted by bursts of EEG activity of 2–3 seconds duration. aEEG trend covering 30 minutes shows a band width of 2.5–7 μV. Sleep–wake cycling is absent

**Figure 6:**
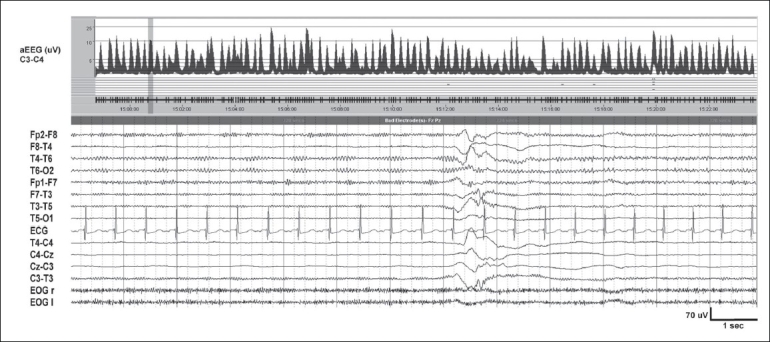
Burst suppression (BS) pattern in severe birth asphyxia. Severely suppressed background activity (<5 μV) with intermittent bursts of 10–20 μV. As opposed to trace discontinu [Figure 5], no EEG activity is discernible during the discontinuous periods. aEEG trend of 30 minutes shows BS and absent variability. Mean band width of the suppressed periods is <2 μV

Abnormalities of voltage seen in encephalopathic neonates, in increasing order of severity are: a) discontinuous EEG [Figure 5], b) continuous low-voltage EEG (voltage < 10 μV throughout the record), c) burst suppression pattern (BS) [Figure 6], and d) an isoelectric EEG or a ‘flat trace’ (voltage consistently < 5 μV).

The discontinuous EEG seen in neonatal encephalopathies like HIE is comparable in morphology to the tracé discontinu seen in prematures. The discontinuous periods tend to be longer[23] and of lower voltage (<10 μV) with increasing severity of brain dysfunction and indicate a poor prognosis.[24–27] BS is the severest form of EEG discontinuity and shows prolonged periods (usually >10 seconds) of marked suppression (voltage consistently <5 μV), interspersed with shorter periods of paroxysmal burst activity and a fairly constant interburst interval (absence of variability). The voltage of the bursts may vary and is generally higher than 50 μV. The content of the bursts is also abnormal, with sharper waveforms and less low-frequency activity.[28] The EEG does not show any reactivity to external stimuli.[29,30] Differentiating severe discontinuity from BS can sometimes be difficult,[23] especially in the premature.The EEG hallmark of severe encephalopathies in older children or adults, that is, diffuse theta/delta activity, is typically not seen in the neonate.

#### Asymmetry:

**Figure 7:**
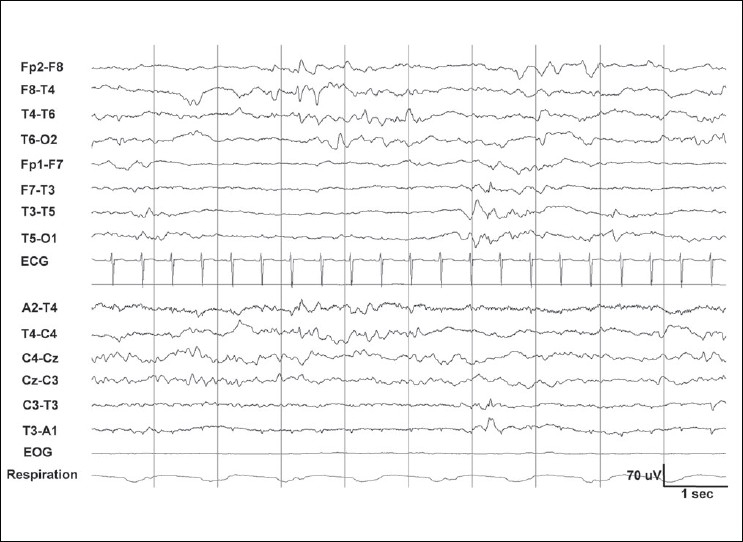
Neonate with a left middle cerebral artery territory infarct. Voltage asymmetry (suppression of EEG activity) over the left temporal region [Figure 8]

Consistent asymmetry in amplitude of >50% between homologous areas of the brain is abnormal.[24] Etiologies include stroke, sinus thrombosis, hemorrhage, and abscess. The abnormal side usually has lower amplitude with often some of the constituents of the normal background activity missing [Figure 7]. This has to be distinguished from a depression of amplitude with preserved background activity, due to technical reasons (shorter interelectrode distances), edema of the scalp, or a subdural collection.

#### Asynchrony:

Persistent asynchrony (bursts of EEG activity occurring >1.5 seconds apart) between homologous areas of both hemispheres, with < 25% of the EEG bursts being synchronous, has been described to be associated with a poor outcome.[24]

#### Effect of medication:

Sedative medications like benzodiazepines and barbiturates transiently affect the background activity in a healthy newborn. However, in a baby with encephalopathy, the effects of these medications on the EEG may be more prolonged, producing attenuation in the voltage of the background activity and producing a discontinuous trace, lasting for up to two hours.[31] When the blood levels of these medications are close to toxic levels, they have a pronounced[32] and sustained effect on the EEG.

#### Paroxysmal activity:

**Figure 8:**
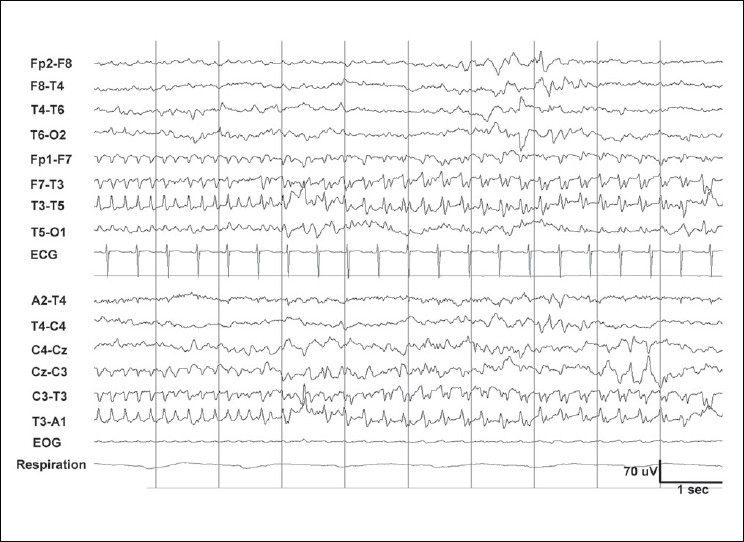
Same patient as in figure 7, shows a seizure over the left temporal region. A frequency of 5 Hz is seen initially. The amplitude then increases, the morphology becomes more complex, and subsequently, the frequency slows down to 3 Hz. This type of evolution is fairly typical for neonatal seizures

**Figure 9:**
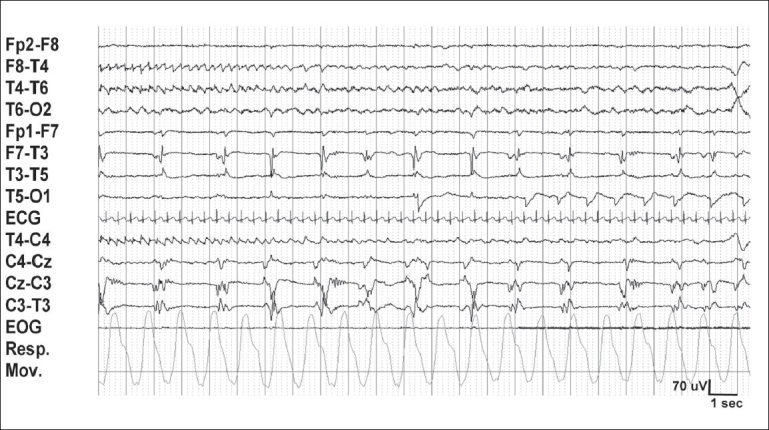
Multifocal seizures on a severely suppressed EEG background in severe birth asphyxia, indicates poor prognosis. The neonate died

Seizures are a common sign of cerebral dysfunction in neonates[33] and are sometimes the only manifestation. A seizure is recognized as a clearcut change in activity from the ongoing background EEG, with repetitive patterns (sharp waves or rhythmic oscillations in delta, theta, or alpha frequencies) lasting for 6–10 seconds. Often, there is a buildup of activity with change in amplitude and frequency [Figures 8, 9]. There is no consensus regarding the length of these discharges.[34] The term brief rhythmic discharges have been used to describe discharges that are shorter than 10 seconds and these probably have the same clinical significance as seizures.[35] The causes of seizures include HIE, metabolic disturbances, intracranial hemorrhages and infarcts, intracranial infection, and developmental anomalies. There is controversy about whether they are just epiphenomena or cause additional brain damage. An increasing number of studies have shown that neonatal seizures are associated with lasting changes in the CNS[36,37] and are related to poor outcome.[38–41] They are further discussed below.

## EEG monitoring in the neonate

EEG monitoring is indicated in neonates with moderate to severe encephalopathy.

### Selection of patients for monitoring

At our center, neonates with the following clinical indications are taken up for continuous monitoring with EEG polygraphy (including video).

1. Neonates with features of severe birth asphyxia: Apgar score ≤5 at 5 minutes, umbilical cord blood pH <7.1.
2. Babies with or without asphyxia but strong clinical suspicion of seizures, like repetitive rhythmic limb movements or unexplained apneas.
3. Neonates with encephalopathy (Sarnat[42] score 2–3), regardless of severity of asphyxia.
4. Critically ill neonates on ventilator, especially if neuromuscular blocking agents are used.
5. If a standard EEG shows moderate to severe abnormalities in the background activity (discontinuity, persistent asymmetry, or presence of epileptiform abnormalities).

If the initial EEG shows a normal or mildly abnormal background activity, continuous EEG monitoring gives little extra information, unless the clinical condition of the neonate subsequently deteriorates or there is suspicion of seizures.

### Aims of monitoring

- Assessment of background activity and detection of changes over time, with the goal of early detection of changes, at a stage when brain dysfunction is potentially reversible. The information can also be used for prognostication.
- Detection of (subclinical) seizures.
- Assessment of response to treatment.

#### Assessment of dynamic changes in background activity (improvement/deterioration):

EEG monitoring is invaluable in assessing the degree and reversibility of brain injury in HIE. Severely abnormal EEG background activity is associated with poor neurological outcome. Persistence of abnormal patterns for 24 hours or more, or worsening of background activity denote poor prognosis.[26,43] However, improvement of the background activity (increasing voltage, decrease in discontinuity, appearance of sleep–wake cycles) within 12–24 hours after birth is a good prognostic feature. Medications[23] such as morphine, phenobarbitone, midazolam, and lidocaine, as well as metabolic or electrolyte disturbances can produce suppression of background EEG voltage, lasting for minutes to hours.

#### Detection of seizures: majority of neonatal seizures are subclinical:

EEG monitoring increases the chance of detecting paroxysmal abnormalities like seizures. Their detection is based on clinical observation in conjunction with (video) EEG. In neonates, the clinical seizures are often subtle[44] and may be missed without constant supervision.[45] Furthermore, in the sick neonate, majority of seizures are subclinical,[46,47] and can be detected only by EEG monitoring.[48,49] A recent study of 51 term neonates with encephalopathy using video EEG monitoring showed that only 34% of the electrographic seizures had clinical manifestations evident on the simultaneous video recording.[50] Only 9% of all electrographic seizures was accompanied by clinical manifestations that were recognized by neonatal staff.[50] Electroclinical dissociation is also common after treatment with antiepileptic drugs (AEDs).[51] All these emphasize the need for continuous EEG monitoring in the encephalopathic neonate. However, EEG analysis requires particular skills which are not always present around the clock in the NICU. Hence, there is scope for an automated system that reliably detects neonatal seizures.

Many seizure detection algorithms have been described.[52–57] The complex nature of the EEG signal results in false-positive seizure detections, especially in methods which show high sensitivity, like that of Liu *et al*.[52] Alternate methods of seizure detection like motion analysis and[58] heart rate analysis[59] have also been explored and were found to be suboptimal.[59] We have recently been involved in developing a seizure detection algorithm mimicking a human observer, with very promising results.[60] Both under and over-diagnosis of neonatal seizures are potentially harmful to the neonate.

#### Clinical utility of EEG monitoring for seizure detection:

In an increasingly cost-conscious medical system, judicious use of EEG monitoring can be done by selecting those neonates with higher risk for developing seizures. This selection may be done using either clinical criteria (e.g., using Sarnat scores for grading encephalopathy[42]) or EEG criteria. Abnormalities of background activity in a standard EEG done within 24 hours of birth is also a good indicator of the risk of seizures [Figures 7 and 8].[61] The degree of suspicion should be high and threshold for starting EEG monitoring low, if more neonates having seizures are to be detected. Posthypoxic seizures usually occur within 24 hours, and may continue for up to 72 hours. In a study of continuous EEG monitoring for neonatal seizures, having more than 25 seizures/day, total daily duration of seizure activity more than 30 minutes, or persistence of seizure activity for more than 48 hours predicted death or major neurological sequelae.[43]

The current practice in most NICUs doing EEG monitoring is to treat subclinical seizures. However, whether this improves the neurological outcome has not yet been proven. Some data[62] suggest a better outcome for treated neonates.

#### Assessment of response to treatment:

Seizures respond to first-line AEDs like phenobarbitone and phenytoin in a relatively small number of patients.[63] Midazolam is the usual second-line AED. Seizures resistant to these may respond to lidocaine. Monitoring helps to determine the response to treatment. In some patients there is a period of initial response[64] followed by recurrence of seizures despite maintaining therapeutic levels of the AED. Seizures that are resistant to multiple AEDs usually suggest a poor neurological outcome.[65]

### Length of EEG monitoring

In the encephalopathic neonate, the yield is maximum if monitoring is started as soon as possible. In conditions like HIE, starting within 24 hours of birth is suggested. However, there is no consensus regarding how long the monitoring should be continued. The following are suggested as reasonable endpoints.

1. Abnormal EEG background improves and normalizes (disappearance of discontinuity, improvement in voltage, return of sleep–wake cycles)
2. Persistently abnormal EEG background even after 24 hours, if other reversible causes like metabolic disturbances have been excluded. This suggests a poor prognosis.
3. No electrographic seizures for at least 12 hours (if monitoring was started for seizures).

### Use of EEG trends for monitoring

Long-term EEG monitoring is labor-intensive. Even in centers where neonatal EEG monitoring is available, around-the-clock support by the clinical neurophysiologist is not possible. This has lead to the development of EEG trends displaying compressed data. Different parameters like aEEG, total power, spectral edge frequency, and entropy have been studied and many of them are included in commercially available EEG equipments. Among these, aEEG has had good acceptance in neonatal ICUs. The following discussion is about the uses and limitations of this technique.

### Amplitude integrated EEG

aEEG (Cerebral Function Monitor, CFM™) displays a simple trend, of the peak-to-peak amplitude derived from a single channel (P3-P4) of EEG. The signal is filtered (band pass 2–15 Hz), rectified, and displayed after compression, with a time base of 6 cm/hour (about 5 hours of EEG data displayed per page). A semi-logarithmic scale is used, that is, EEG activity of lower amplitudes (0–10 μV) which is of maximal interest to the clinician, is displayed on a linear scale while the higher amplitudes (10–100 μV) appear more compressed (logarithmic scale). In the healthy neonate, the aEEG band runs between 10 and 20 μV, with fluctuations in voltage reflecting changes in state. Sleep–wake cycling can readily be recognized [Figures 3 and 4].

### Background activity

Al Naqeeb *et al*,[66] classified the aEEG background into three groups in the full-term neonate using different voltage cut-offs for the median upper (UM) and lower margins (LM): Normal (UM > 10 μV, LM > 5 μV), moderately abnormal (UM > 10 μV, LM < 5 μV), and suppressed (UM < 10 μV, LM < 5 μV). Another classification of aEEG in the term neonate, based more on pattern recognition than absolute amplitudes, recognizes five major patterns:[67–70] continuous normal voltage (CNV, band 25–10 μV), discontinuous normal voltage (DNV, UM > 10 μV, LM < 5 μV), continuous low voltage (CLV, band ≤ 5 μV), burst suppression (BS), and flat trace (FT, UM < 5 μV). This classification is more detailed, and more studies have been published based on this. In term neonates with postasphyxial HIE, the CNV and DNV at six hours correlated with a good outcome while CLV, BS, and FT predicted poor outcome.[68] Classification of background activity by aEEG was shown to have a good agreement with raw EEG.[71]

### Detection of seizures

aEEG has moderate sensitivity for detecting seizures.[71–73] They are seen as an abrupt increase in voltage of the upper and lower margins of the trace, along with a narrowing of the band. Seizures in the centroparietal region are readily detected. Different seizure patterns described by aEEG include single seizure, repetitive seizures, and status epilepticus (the so-called saw-tooth pattern).[71]

### Advantages and limitations of aEEG

aEEG scores over full-channel raw EEG by its ease of registration and interpretation and is suited for long-term monitoring by the bedside. It is ideal for observing long-term trends in the brain function like improvement or deterioration of background activity voltage, effect of medications, occurrence of seizures, etc.[69,74]

However, due to the use of only a single EEG channel, important spatial information is lost. Thus, asymmetries as well as seizure activity may be missed. The data compression sometimes results in incorrect estimation of the background activity voltage and missing of seizures that are very focal, of short duration, or of low voltage. Also, contamination by artifacts like ECG, muscle activity, or high-frequency ventilation may result in falsely elevated aEEG voltage and sometimes lead to false-positive ‘seizure detection’.[75,76]

Many aEEG monitors also display corresponding single channel of raw EEG, and this helps in improving the quality of interpretation, like recognizing artifacts and confirming occurrence of paroxysmal activity like seizures. aEEG is best viewed as being complimentary to standard EEG. Whenever major therapeutic decisions have to be taken (e.g., when recurrent seizures are seen and an AED infusion is being considered), it is advisable to review the corresponding raw EEG data if available, or record a standard EEG.

## Estimating prognosis using EEG

Both EEG and aEEG background activity has been correlated well with neurological outcome. A normal background activity is strongly correlated with good outcome in term neonates. Also, a severely abnormal background activity (persistent low voltage, inactive record, as well as an unvarying BS pattern) is correlated with poor outcome.[77] The background patterns occurring between these two extremes are more difficult to use in outcome predictions, with different studies giving varying results. In such situations, clinical assessment coupled with serial EEGs or EEG monitoring, as well as multimodal evaluation using neuroimaging (ultrasonogram, MRI), and evoked potentials (SEPs, VEPs)[78,79] would help in prognostication. Persistence of the abnormal patterns in the EEG has more prognostic value [Figure 9].[80] Assessing the prognosis in prematures is more difficult as comorbidities like pulmonary problems may determine the outcome.

## Neonatal Epilepsy

Though most neonatal seizures are acute symptomatic (provoked) in nature, a few epilepsy syndromes can manifest in this period. Some are benign while others have a grave prognosis. It is beyond the scope of this article to describe various neonatal epilepsy syndromes. However, some of these rare syndromes with characteristic EEG features are described below as it is important to differentiate them from acute symptomatic seizures. Majority of these have a poor neurological outcome.

1. *Pyridoxine dependency*: Presents with myoclonic and tonic-clonic seizures with or without an associated encephalopathy. EEG findings include generalized high amplitude slowing of the background activity, multifocal or generalized epileptiform abnormalities, or in severe cases, a BS pattern.[81] A high degree of clinical suspicion is needed to diagnose this condition and early treatment with pyridoxine is indicated.
2. *Herpes simplex encephalitis*: Presents in the neonatal period with fever, seizures, and obtundation. EEG findings include generalized or asymmetric slowing of the background activity, and periodic lateralized epileptiform discharges.[82]
3. *Nonketotic hyperglycinemia* can present in the neonate with refractory seizures and a BS pattern.[83]
4. *Ohtahara syndrome (Early infantile epileptic encephalopathy or EIEE) and early myoclonic encephalopathy (EME)*: Both can show a BS pattern on EEG. The BS (sometimes asymmetric) in EIEE is present in both wakefulness and sleep and persists unchanged for about two weeks, while in EME, it is seen mainly in sleep.[84] EIEE is caused by cortical malformations or migrational disorders, and presents with refractory tonic seizures while EME is caused by inborn errors of metabolism and usually presents with myoclonic seizures.

## Conclusion

Neonatal EEG gives important information about brain maturation and function and complements clinical examination. It also helps in detecting seizures and in prognostication.

- The technician should note the CA and clinical setting of EEG registration.
- Polygraphy helps in differentiating awake and sleep states and in recognizing artifacts.
- EEG is classified as normal/abnormal for CA. Reviewing at a time base of 20 seconds/page makes detection of slow activity and periodic or rhythmic phenomena easier. Abnormalities are classified as mild, moderate, and severe.
- Mild abnormalities include dysmaturity, excessive sporadic sharp transients, etc.
- Moderate abnormalities include abnormal or absent sleep–wake cycles, excessive discontinuity, persistent asymmetry, and epileptiform abnormalities including seizures.
- Severe abnormalities include persistent low voltage, BS, and inactive/isoelectric EEG.
- When moderate or severe EEG abnormalities are detected, EEG monitoring can give additional information regarding the evolution of background activity and the occurrence of seizures.
- EEG trends like aEEG are useful for the bedside monitoring of sick neonates, provided its benefits and limitations are well understood.
